# Post-parotidectomy facial nerve function: comparison between original and modified Sunnybrook Facial Grading Systems

**DOI:** 10.1055/s-0043-1777003

**Published:** 2023-11-30

**Authors:** Márcia Gonçalves e Silva Targino da Costa, Péricles de Andrade Maranhão-Filho, Izabella Costa Santos, Carolina Rocha Aquino González, Carlos Henrique Stohler de Almeida, Ronir Raggio Luiz

**Affiliations:** 1 Hospital do Câncer I, Instituto Nacional de Câncer, Departamento de Fisioterapia, Rio de Janeiro RJ, Brazil.; 2 Universidade Federal do Rio de Janeiro, Hospital Universitário Clementino Fraga Filho, Departamento de Neurologia, Rio de Janeiro RJ, Brazil.; 3 Hospital do Câncer I, Instituto Nacional de Câncer, Departamento de Cirurgia de Cabeça e Pescoço, Rio de Janeiro RJ, Brazil.; 4 Universidade Federal do Rio de Janeiro, Instituto de Estudos em Saúde Coletiva, Rio de Janeiro RJ, Brazil.

**Keywords:** Parotid Neoplasms, Skin Neoplasms, Surgery, Facial Nerve Injuries, Patient Outcomes Assessment., Neoplasias Parotídeas, Neoplasias Cutâneas, Cirurgia, Traumatismos do Nervo Facial, Avaliação de Resultados da Assistência ao Paciente.

## Abstract

**Background:** Facial nerve dysfunction is the principal postoperative complication related to parotidectomy.

**Objective:** To test the hypothesis that the modified Sunnybrook Facial Grading System (mS-FGS) is superior to the original S-FGS in the assessment of facial nerve function following parotidectomy.

**Methods:** Prospective, longitudinal study evaluating patients with primary or metastatic parotid neoplasms undergoing parotidectomy with facial nerve-sparing between 2016 and 2020. The subjects were assessed twice, on the first postoperative day and at the first outpatient evaluation, 20-30 days post-surgery. Facial assessments were performed using the original and modified (plus showing the lower teeth) versions of the Sunnybrook System and documented by pictures and video recordings. Intra- and inter-rater agreements regarding the assessment of the new expression were analyzed.

**Results:** 101 patients were enrolled. In both steps, the results from the mS-FGS were significantly lower (p < 0.001). Subjects with a history of previous parotidectomy and those who underwent neck dissection had more severe facial nerve impairment. The mandibular marginal branch was the most frequently injured, affecting 68.3% of the patients on the first postoperative day and 52.5% on the first outpatient evaluation. Twenty patients (19.8%) presented an exclusive marginal mandibular branch lesion. The inter-rater agreement of the new expression assessment ranged from substantial to almost perfect. The intra-rater agreement was almost perfect (wk = 0.951).

**Conclusion:** The adoption of the Modified Sunnybrook System, which includes evaluation of the mandibular marginal branch, increases the accuracy of post-parotidectomy facial nerve dysfunction appraisal.

## Resumo

**Antecedentes:** A disfunção do nervo facial é a principal complicação pós-operatória relacionada à parotidectomia.

**Objetivo:** Testar a hipótese de que o sistema Sunnybrook de graduação facial modificado (mS-FGS) é superior ao S-FGS original na avaliação da função do nervo facial após parotidectomia.

**Métodos:** Estudo longitudinal prospectivo avaliando o pós-operatório de pacientes com neoplasias parotídeas primárias ou metastáticas, submetidos à parotidectomia com preservação do nervo facial, entre 2016 e 2020. Os indivíduos foram avaliados duas vezes, no primeiro dia de pós-operatório e na primeira avaliação ambulatorial, 20-30 dias após a cirurgia. As avaliações faciais foram realizadas usando as versões original e modificada (que incluem mostrar os dentes inferiores) do sistema Sunnybrook e documentadas por fotos e vídeos. Foram adicionalmente analisadas as concordâncias intra e interexaminadoras da avaliação da nova expressão.

**Resultados:** Cento e um pacientes foram incluídos. Em ambas as etapas, os resultados do mS-FGS foram significativamente menores (p < 0,001). Indivíduos com história de parotidectomia prévia e aqueles submetidos ao esvaziamento cervical apresentaram comprometimento mais grave do nervo facial. O ramo marginal mandibular foi o mais afetado, acometendo 68,3% dos pacientes no primeiro dia de pós-operatório e 52,5% na primeira avaliação ambulatorial. Vinte pacientes (19,8%) apresentaram lesão exclusiva do ramo marginal mandibular. A concordância interexaminadores da avaliação da nova expressão variou de substancial a quase perfeita. A concordância intraexaminador foi quase perfeita (wk = 0,951).

**Conclusão:** A adoção do sistema Sunnybrook modificado, que inclui a análise do ramo marginal mandibular, aumenta a precisão da avaliação da disfunção do nervo facial pós-parotidectomia.

## INTRODUCTION

Facial nerve (FN) dysfunction is the main early parotidectomy postoperative complication. 1 The FN injuries occur within the parotid gland, either in the trunk or in each of its branches, leading to various degrees of FN impairment. The mandibular marginal branch (MMB) is the most affected FN segment following parotidectomy. 2 3 4 5 The MMB dysfunction results in the inability to move the lower lip downwards and laterally, and to show the lower teeth, causing asymmetric smiling and mouth opening. This dysfunction is evident predominantly with crying. 6

Ideally, an instrument for facial function assessment must be able to analyze all FN branches and distinguish nuances in severity. In 2015, the Sunnybrook Facial Grading System (S-FGS) was indicated as the gold standard in addressing FN disorders. 7 8 Despite being a widely accepted instrument, the S-FGS does not include the assessment of muscles predominantly innervated by the MMB.

A modified S-FGS version (mS-FGS), focusing specifically postparotidectomy patients, which includes the assessment of the MMB function, was proposed in 2019. 9 In the mS-FGS, the command “snarl”, performed by the *levator labii superioris alaeque nasi and levator labii superioris* (LLA/LLS) muscles, was replaced by “show the lower teeth”, performed by the *depressor labii inferioris and depressor anguli oris* (DLI/DAO) *muscles* . The LLA/LLS are innervated by the zygomatic and buccal branches, 10 which are already assessed by other expressions in the original instrument (eye closure, smile, and lip pucker). On the other hand, the DLI/DAO muscles are innervated by the MMB, a branch not evaluated by any other expression in the S-FGS. 6 9

Several authors have demonstrated a good correlation between the intra- and inter-rater reliability of the S-FGS, with both experienced and younger users. 11 12 13 However, the intra and inter-rater reliability of the new expression evaluation has not been tested.

The objective of this study was to test the hypothesis that the mS-FGS (which includes the MMB) is superior to the S-FGS in the assessment of the facial nerve after parotidectomy due to neoplasms. Furthermore, analyze the intra- and inter-rater reliability of the new expression evaluation.

## METHODS

This was a prospective longitudinal study. Patients who underwent parotidectomy with FN sparing, due to primary or metastatic parotid neoplasms, in a reference oncological institute between February 2016 and February 2020 were sequentially enrolled. Exclusion criteria were previous FN dysfunction, resection of facial expressions muscles during surgery, cognitive impairment, patients younger than 18 years, and refusal to participate in the study.

### Procedures

The main author, a senior physical therapist with 15 years of experience in the rehabilitation of patients with head and neck cancer, performed all assessments. On the 1st postoperative day (POD 1), subjects were evaluated according to the current protocol at the Physiotherapy Department, routinely used since 2006. The protocol is composed of the Original Sunnybrook System, 7 with the addition of the expression “show the lower teeth” (by the same grading methodology), aiming at the assessment of the marginal mandibular branch. From this evaluation protocol, the scores of both systems (original and modified) were calculated.

The S-FGS 7 encompasses 3 parts:

- The Resting Symmetry Score (RSS): evaluates key points of the face at rest. The sum of the results is multiplied by 5;
- The Voluntary Movement Score (VMS): classifies the voluntary movement of 5 key expressions: forehead wrinkle, eye closure, smile, snarl, and lip pucker. Each expression is graded from 1 to 5: 1 = absence of movement; 2 = initiate slight movement; 3 = initiate movement with mild excursion, 4 = movement almost complete, and 5 = complete movement. For the mS-FGS, 9 the “snarl” expression was replaced by “show the lower teeth”. The sum of the results is multiplied by 4;
- The Synkinesis Score (SS) classifies the presence and intensity of synkinesis during each expression.

The result is a Composite Score (CS) calculated as CS = VMS - RSS - SS. Values were calculated from both systems (S-FGS and mS-FGS). All facial assessments were documented with digital pictures and video recordings (iPhone 6S Plus – Apple Inc).

Patients who had some FN dysfunction received an illustrated brochure with tailored facial exercises, according to the affected expressions. They were trained to perform the exercises after hospital discharge (10 repetitions, 3 times a day, with mirror feedback). 14

Then, the participants were scheduled for the first outpatient physiotherapeutic evaluation, performed after the acute healing period, about 20-30 days after surgery (POD 20-30). At this appointment, patients were reassessed by the same examiner using the same methodology.

For intra-rater agreement, the recorded videos were watched and analyzed two times by the main researcher (rater B), who is the most experienced examiner (15 years). The interval time between the two video analyses was six months. For inter-rater agreement, the photos and videos were watched and analyzed by the main researcher and two other physical therapists (raters A and C). Rater A had 5 years of experience in the rehabilitation of patients with head and neck cancer, and rater C had 7 years. The examiners had no time limit to evaluate each patient and could evaluate the videos more than once, if necessary. Each observer performed picture and video evaluations without knowing the other examiners' impressions. In addition, intermethod agreement, which was the correlation between live and video exams, performed by the main researcher, was evaluated. The time interval between the live examination and the evaluation of the photos and videos was at least 12 months.

The Mann-Whitney and Kruskall-Wallis non-parametric tests were used to contrast the scores (original and modified) with categorical variables. The Wilcoxon test was used to analyze the difference between the original and modified S-FGS. The weighted Kappa coefficient was used for the analysis of intra- and inter-rater agreement, considering the interpretation proposed by Landis and Koch. 15 The analyses were performed in SPSS software. Statistically significant results were considered at p values < 0.05.

This study was approved by the National Cancer Institute Research Ethics Committee under registration number 49889015.0.0000.5274, in October 2015. Informed consent was obtained from all participants, including permission to publish their photographs in scientific disclosures.

## RESULTS

During the study, 142 patients meeting enrollment criteria underwent parotidectomy with facial nerve sparing. Forty-one were excluded because of resection of facial expression muscles (10); previous FN dysfunction (8); being younger than 18 years (6); cognitive deficit (4); refused (9); did not attend the first outpatient evaluation (3); and other (1). The final sample consisted of 101 patients: 49 women (48.5%), with a mean age of 54.5 years (21-86). The sample characteristics are described in Table 1 .

**Table 1:** Sample characteristics

		Average (min-max) or N (%)			
Sample characteristics		Total	Benign parotid	Malignant parotid	Metastatic
Participants		101 (100)	69 (68.3)	15 (14.9)	17 (16.8)
Age, years		54.5 (21-86)	52.9 (23-85)	53.2 (21-86)	62.2 (33-81)
≥ 60 years old		43 (42.6)	29 (42.0)	3 (20)	11 (64.7)
Gender, female		49 (48.5)	35 (50.7)	11 (73.3)	3 (17.6)
Type of parotidectomy	Superficial/partial	92 (91.1)	63 (91.3)	13 (86.7)	16 (94.1)
	Subtotal/total	9 (8.9)	6 (8.7)	2 (13.3)	1 (5.9)
Resection of other structures		20 (19.8)	5 (7.2)	3 (20)	12 (70.6)
Neck dissection		23 (22.8)	6 (8,7)	5 (33.3)	12 (70.6)
Previous parotidectomy		8 (7.9)	7 (10.1)	1 (6.7)	0
Reconstruction		8 (7.9)	0	0	8 (47.1)

Most surgeries were for benign neoplasms (68.3%). In this group, 5 patients underwent resection of other structures: thyroid gland (2), paraganglioma (1), skin segments (1), and preparotid lymph node plus accessory gland (1). Regarding the 17 cases of metastatic diseases (16.8%), 15 were skin cancers and 2 were conjunctival melanomas.

The facial evaluations performed on the 1st and between POD 20-30 revealed scores depicted in Table 2 . In both stages, the Voluntary Movement Score and the Composite Score were significantly lower when using the mS-FGS (p *<* 0.001). The Resting Symmetry Score remained unchanged as it did not present any modifications. The Synkinesis Score was zero for both instruments, as they are not expected within the first 30 days after the injury.

**Table 2:** Facial Scores on the POD 1 and POD 20-30

		Mdn (Q1-Q3)		*p* value
Time	Scores	S-FGS	mS-FGS	≠ (S-FGS – mS-FGS)
POD 1	RSS	5 (0 - 10)	5 (0 - 10)	
	VMS	84 (60 - 96)	76 (60–88)	< 0.001
	SS	0	0	
	CS	80 (49.5 - 92)	69 (46.5–86)	< 0.001
POD 20-30	RSS	5 (0 - 10)	5 (0 - 10)	
	VMS	92 (66 - 96)	80 (64 - 92)	< 0.001
	SS	0	0	
	CS	87 (55 - 96)	76 (51 - 92)	< 0.001

Abbreviations: CS, Composite Score; Mdn, Median; POD, Postoperative day; RS, Resting Symmetry Score; SS, Synkinesis Score; VMS, Voluntary Movement Score.

The MMB was the most affected branch. On the 1 ^st^ POD, the worst weakness (degrees 1 to 3) of the DLI/DAO muscles was seen in 60.9% of patients with benign parotid neoplasms, 73.3% of those with parotid cancer, and 94.1% of the cases with metastatic disease. Concerning the total sample, MMB dysfunction was evidenced in 68.3% of the subjects.

**Figure 1:**
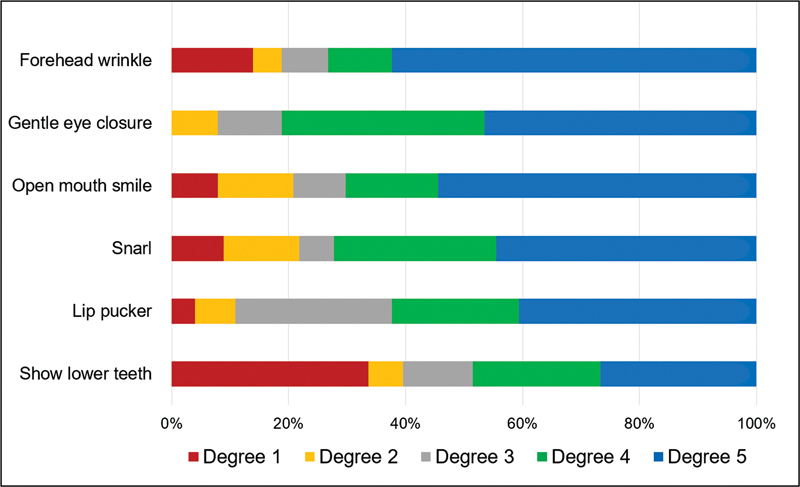
Degree of voluntary movement of each expression assessed on POD 20-30.

**Figure 2:**
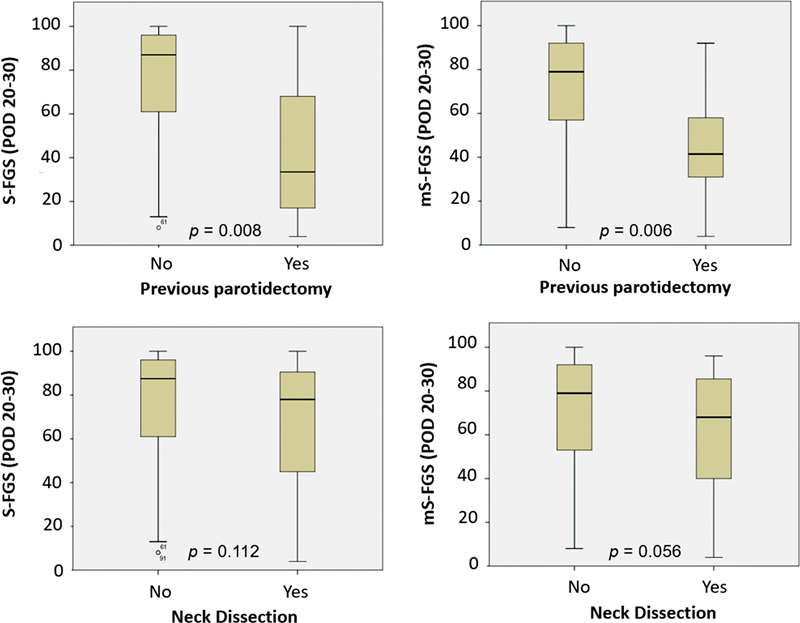
Graphic representation of the statistically significant results in comparison of S-FGS and mS-FGS with clinical-surgical variables (POD 20-30).

The POD 20-30 assessment was performed, on average, on the 25.9 ± 6.8 day. Figure 1 shows the degree of voluntary movement for each expression evaluated at POD 20-30. Subjects with a history of previous parotidectomy had more severe facial dysfunctions and it was evidenced by both S-FGS and m-SFGS (p = 0.008 and p = 0.006, respectively), as exposed in Figure 2 . Neck dissection was also responsible for worse FN impairment, but it was noticeable only by the mS-FGS (p = 0.056). At the 20-30 POD, the DLI/DAO dysfunction remained in 52.5% of cases.

**Figure 3:**
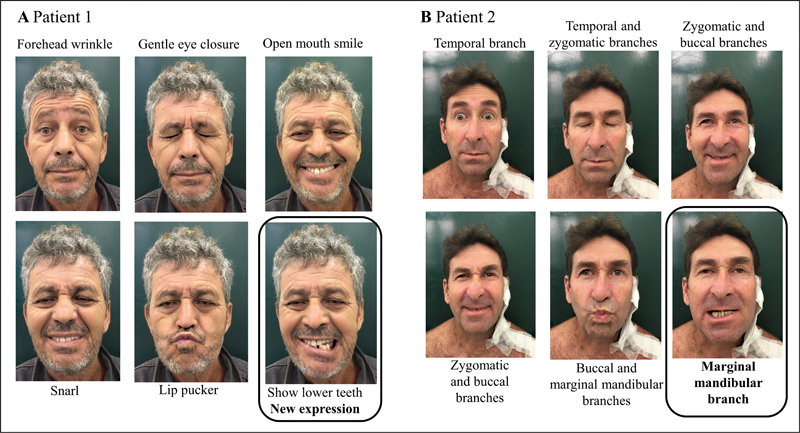
Examples of exclusive MMB dysfunction post parotidectomy (POD 20-30). A Patient 1: 54-year-old man after superficial parotidectomy due to a Warthin Tumor. The first 5 images correspond to expressions evaluated by the S-FGS. The sixth picture (highlighted) shows the new expression introduced in mS-FGS. B Patient 2: 49-year-old man after more aggressive surgery: resection of retroauricular skin cancer plus superficial parotidectomy, neck dissection (levels II and III) and reconstruction with SCAIF flap, due to squamous cell carcinoma. The pictures present the same expressions, also identifying the corresponding predominant branches of the facial nerve under test. The patient had mild OO paresis but a DLI/DAO palsy.

An exclusive MMB lesion was identified in 20 patients (19.8%). Ten of them had only DLI/DAO dysfunction (9.9%). The other 10 patients had concomitant mild paresis (grades 3 or 4) of the orbicularis oris (OO) muscle. However, DLI/DAO impairment was severe in these last patients, with paralysis (grade 1) seen in 7 of the 10 cases. Figure 3 shows two examples of patients with exclusive MMB dysfunction.

Only 4 patients had exclusive paresis of the “snarl” expression; however, the dysfunctions were mild (degree 4: almost complete movement).

The comparison between the movement degrees of the “snarl” and “show the lower teeth” expressions on POD 20-30 is shown in Table 3 . The comparison between the Voluntary Movement Scores of the S-FGS and the mS-FGS revealed the lowest scores obtained by the modified system (p < 0.001), according to the following distribution:

**Table 3:** Comparison between the degree of movement of the "snarl" and "show the lower teeth" expressions on POD 20-30

	Show the Lower Teeth (POD 20-30)					
Snarl (POD 20-30)	No movement	Initiates slight movement	Initiated movement with mild excursion	Movement almost complete	Movement complete	Total
No movement	4	0	0	3	2	9
Initiates slight movement	5	0	5	3	0	13
Initiated movement with mild excursion	5	0	0	0	1	6
Movement almost complete	7	2	2	7	10	28
Movement complete	14	4	5	9	13	45
Total	35	6	12	22	26	101

Note: In the diagonally highlighted rectangle are the 24 cases of mS-FGS = S-FGS. Below the rectangle are the 53 cases of mS-FGS < S-FGS. Above the rectangle are the 24 cases of mS-FGS > S-FGS.

- mS-FGS = S-FGS: 24 cases;
- mS-FGS > S-FGS: 24 cases;
- mS-FGS < S-FGS: 53 cases.

The analysis of the interrater and intra-rater agreement of the “show lower teeth” expression evaluation involved 100 patients, as images of one patient were not recorded. The interrater agreement among the three evaluators (A, B, and C) ranged from substantial to almost perfect ( Table 4 ).

**Table 4:** Interrater agreement for the video evaluation of the expression "show the lower teeth" for three raters (A, B, and C)

	Degrees		1	2	3	4	5	Weighted kappa
	Rater B							
**Inter-rater**	**1**	Rater A	12	4	0	0	0	***wk*** **=** **0.698**
	**2**		12	4	0	0	0	
	**3**		3	8	3	1	0	
	**4**		0	1	7	4	0	
	**5**		0	0	1	12	28	
	Rater C								
	**1**	Rater B	21	6	0	0	0	***wk*** **=** **0.833**
	**2**		7	5	5	0	0	
	**3**		0	1	7	3	0	
	**4**		0	0	1	11	5	
	**5**		0	0	0	2	26	
	Rater C								
	**1**	Rater A	13	2	1	0	0	***wk*** **=** **0.742**
	**2**		11	4	1	0	0	
	**3**		4	5	6	0	0	
	**4**		0	1	5	6	0	
	**5**		0	0	0	10	31	

In 11 cases, an expressive difference of 2 degrees was identified between the results of the raters. Five of them occurred in the comparison between the less experienced examiner (A) and the most experienced (B). The other 6 occurred between the less experienced and the one with intermediate experience (C).

The intra-rater agreement of the video assessments was almost perfect (wk = 0.950). An almost perfect agreement (wk = 0.878) was also observed in the intermethod analysis (between live and video appraisal), as shown in Table 5 .

**Table 5:** Intra-rater and inter-method agreement for the evaluation of the expression "show the lower teeth" (rater B)

	Degrees		1	2	3	4	5	Weighted kappa
**Intra-rater**	Video 2							
	**1**	Video 1	26	1	0	0	0	***wk*** **=** **0.950**
	**2**		1	14	2	0	0	
	**3**		0	0	7	4	0	
	**4**		0	0	0	16	1	
	**5**		0	0	0	0	28	
**Inter-method**	Video 1							
	**1**	Live	26	9	0	0	0	***wk*** **=** **0.878**
	**2**		1	4	1	0	0	
	**3**		0	4	7	1	0	
	**4**		0	0	3	16	3	
	**5**		0	0	0	0	25	

Notes: Video 1: First evaluation by video; Video 2: Second evaluation by video.

## DISCUSSION

The present results show that the mS-FGS is more sensitive to detect the magnitude of the parotidectomy impact on the FN function involving different branches as it allows the examination of the face as a whole. This is supported by the significantly lower scores obtained with the mS-FGS as compared to the S-FGS in both the immediate and post ∼30 days postoperative period assessments.

Primary parotid neoplasms represent 1% to 3% of head and neck (HN) tumors. 16 Skin cancer is the most common form of cancer, and its incidence has been growing. 17 About 75% to 90% of all cutaneous squamous cell carcinomas occur in the HN area, especially in the face, due to increased exposure to solar radiation. 18 As for cutaneous melanomas, it is estimated that 6% to 25% of lesions occur in the HN. 19 Parotid lymph nodes are common sites of metastases from advanced skin cancers that affect the HN. 20 Parotidectomy (superficial or total), associated with neck dissection, must be considered in all patients with intraparotid and cervical lymph node metastases. 21 In this epidemiological context, a large number of patients need an accurate facial appraisal after surgery.

Considering the frequency of MMB injuries during parotidectomies, the FN grading instrument must include the muscles predominantly innervated by this branch. Since the S-FGS (considered the gold standard) does not include the MMB, the DLI/DAO assessment needs to be recorded separately. The consequence is an underestimated S-FGS score, which often does not reflect the total extent of facial dysfunction. Parallel to the improvement of parotidectomy techniques to mitigate the damage to the MMB, it is also necessary to refine the postoperative clinical examination of the FN. 22

In our series, the MMB was the most affected branch in all subgroups. The dysfunction was present in more than 2/3 of the patients on POD 1 and just over half at the first outpatient facial evaluation, showing some degree of recovery in the first ∼30 days. Several other studies evidenced a predominant involvement of the MMB in patients with FN dysfunction after parotidectomy. 3 4 5 23 This is the most frequent complication in surgeries for benign neoplasms. 24 Infante-Cossio et al. reported that the MMB was the most affected (64.5% of the patients) in the first week after surgery for pleomorphic adenoma. 5 Musani et al. reported that the MMB was involved in 57 (86.3%) of the 66 cases who had immediate postoperative FN dysfunction in their study about FN morbidity following surgery for benign tumors. 25 A similarly high percentage of MMB weakness was described by Hwang and Brett, 26 present in 92.3% of the patients who presented postoperative FN dysfunction. Gaillard et al. reported 39% of MMB impairment after surgeries for benign and malignant parotid neoplasms. 4

We had 20 cases of exclusive MMB lesions. In 10 of them, the single manifestation was DLI/DAO dysfunction, which would not be detectable by the original S-FGS. Even in the other 10 cases with some weakness in the *orbicularis oris* (OO), the dysfunction of this muscle was mild (degrees 3 and 4), not reflecting the real intensity of the MMB impairment. Our findings regarding the impact of exclusive MMB lesions are consistent with the study by Raslan et al. 27 who reported that, during parotidectomy, electrical stimulation of the cervicofacial division (MMB and cervical branch) of the facial nerve was always followed by movement of the mouth and chin region, related to the OO and the DAO muscles. This suggests that the OO can also receive supply from the MMB, but the impairment in this muscle was clinically visible in only half of the cases of exclusive MMB injury in our sample. Isolated MMB lesions following parotidectomy were also reported by other authors. 3 4 28 In many cases, this is the unique expression of FN disorder after surgery. In addition to cosmetic deformity, common complaints related to exclusive MMB weakness include decreased oral continence and lower lip biting during feeding.

Subjects with a history of previous parotidectomy had more severe FN dysfunctions. Seven of the 8 cases had pleomorphic adenoma, a neoplasm with a high rate of recurrence. The reoperations increase the risk of nerve damage due to the difficulty in distinguishing the FN from scar tissue and fibrosis. 29 In parotidectomy due to recurrent tumors, intraoperative FN monitoring may result in less severe injuries and faster recovery. 30

Patients who needed neck dissection also had worse FN impairment, although it was only perceptible by the mS-FGS, which evaluates the MMB. Neck dissection is an isolated risk factor for MMB injury, 6 31 especially at the levels Ib and IIa of cervical lymph nodes. 32 The procedure adds significant morbidity to the MMB during parotidectomy. Bron and O'Brien emphasized that every effort is necessary to minimize the risk of MMB injury in parotidectomy with neck dissection. 33 However, they also highlighted the risks involved with the challenging removal of lymph nodes related to the facial vessels and lower edge of the mandible, an area crossed by this thin and delicate facial nerve branch. The morbidity of neck dissection to the FN in parotidectomies also was highlighted by Eviston et al. 34 They reported 3.5 times the odds of facial palsy compared with those patients who did not undergo the procedure. 34

Since the mS-FGS initial proposal was based on a retrospective study, the intra- and interrater reliability of the new expression (show lower teeth) assessment had not yet been tested. In the present study, we found interrater reliability ranging from substantial to almost perfect. Regarding the results of the other expressions contained in the S-FGS, Cabrol et al. reported an interrater agreement almost perfect for forehead wrinkles and open-mouth smile; and important (or substantial) for gentle eye closure, snarl, and lip pucker. Delphine et al., studying post-parotidectomy FN assessments, also found good to excellent interrater reliability in the S-FGS Voluntary Movement Score. 35 In our sample, the greatest discordance was observed between degrees 1 and 2 (absence of movement and slight movement). When reassessing the 11 cases with expressive differences of 2 degrees between the raters' results, we found that the greatest discrepancies came from the less experienced examiner (rater A). This finding suggests that the degree of experience can impact the assessment's precision. It is consistent with the report that the interrater agreement of the S-FGS by inexperienced observers gradually improved over time, plateauing after ∼70 evaluations. 36

The intra-rater agreement of the new expression was almost perfect, being equivalent to that reported by Cabrol et al. in the evaluation of the 5 standard expressions. In terms of intermethod reliability, the agreement between video and live assessment of the DLI/DAO muscles also was almost perfect. Our findings are consistent with those reported by Tan et al. in their study comparing face-to-face versus video assessment of facial paralysis using 3 different instruments. 37 Regarding the S-FGS, the authors reported that the reliability was good to excellent when assessing voluntary movement. The agreement also ranged from good to excellent across all parameters for both live and video assessments. In our sample, a trend towards higher movement degrees was observed in evaluations by video. This result may be linked to the possibility of reviewing the videos several times and identifying nuances of movement that were less obvious during the live assessments. The discrepancies were more frequent between degrees 2 and 3 (slight movement and movement with mild excursion).

Our study has limitations. The low number of patients, especially in parotid cancer and skin cancer subgroups, despite the long inclusion period, and the expressive number of exclusions might have impacted our results. The profile of our sample can be justified by the lower prevalence of parotid cancer and the need for partial or total resection of the FN in more advanced cases. 38 Despite the high prevalence of skin cancer, surgeries involving parotidectomy tend to be more aggressive, often including the FN and/or facial muscles in the resection. 39 Another limitation was the reduced number of examiners for the interrater reliability analysis. Additionally, it is important to emphasize that the possibility of anatomical variations in the distribution of the branches of the FN, 40 as well as the relatively rare agenesis of facial muscles can impact the results of the facial examination. 41

In conclusion, our results demonstrate that the replacement of S-FGS with mS-FGS significantly improves the precision of the facial nerve assessment after parotidectomy. The replacement of the “snarl” with the “show the lower teeth” expression, performed by muscles innervated by the MMB, makes the instrument more sensitive to identifying typical nuances of FN disorders after these surgeries. The intra-rater agreement of the new expression assessments was almost perfect, and the interrater agreement ranged from substantial to almost perfect.
